# Dynamic Compression Mechanical Behavior of Three-Dimensional Chiral Mechanical Metamaterials: Effects of Geometric Parameters and Size

**DOI:** 10.3390/ma18112584

**Published:** 2025-06-01

**Authors:** Shidi Qin, Zhenyang Huang, Weidong Cao, Xiaofei Cao, Yongshui Lin

**Affiliations:** 1 Hubei Key Laboratory of Theory and Application of Advanced Materials Mechanics, Department of Engineering Mechanics, School of Physics and Mechanics, Wuhan University of Technology, Wuhan 430070, China; shidiqin_lixue@whut.edu.cn (S.Q.); zhenyanghuang_lixue@whut.edu.cn (Z.H.); peakspylin@163.com (Y.L.); 2 Research Center of Fluid Machinery Engineering and Technology, Jiangsu University, Zhenjiang 212013, China; cwd@ujs.edu.cn

**Keywords:** three-dimensional chiral mechanical metamaterials, dynamic compression behavior, size effect, compression–torsion coupling effect

## Abstract

The coupled compression–torsion effects of three-dimensional chiral mechanical metamaterials have attracted widespread attention from researchers in recent years. However, the deformation rules and mechanisms through which geometric parameters and size affect their quasi-static and low-speed dynamic compression behavior are still unclear. This paper numerically investigates the quasi-static and low-speed dynamic compression mechanical behavior of three-dimensional chiral mechanical metamaterials, and the effects of geometric parameters and size are discussed. The numerical results are validated by their comparison with the experimental data. The testing results indicate that the geometric parameters as well as number of arrays in different directions have a significant effect on the quasi-static and dynamic compression twist angle per axial strain and the effective modulus. Interestingly, the values of the twist angle per axial strain under static and dynamic compression are almost the same under the same strain, but the effective modulus decreases more sharply under dynamic loading conditions, which may be due to inertia. Our work elucidates the mechanism through which geometric parameters and size affect the quasi-static and dynamic deformation behavior of three-dimensional chiral mechanical metamaterials, which provides design references for their practical engineering applications.

## 1. Introduction

Mechanical metamaterials are a class of materials with specific mechanical properties that are produced by designing their internal microstructures. The unique properties of these materials are not derived from the materials they are composed of but rather from their structural design. These metamaterials exhibit many unique mechanical properties that do not exist in nature, achieved through the ingenious design of geometry, arrangement, and topology. The application scenarios of mechanical metamaterials are extensive. Progress has been made in the fields of mechanics [1,2,3], acoustics [4,5,6,7], optics [8,9,10,11], energy absorption [12,13,14], biomaterials [15], robotics [16,17,18,19], and 3D material printing [20,21,22]. These mechanical metamaterials have been applied for various purposes in these fields by using their negative Poisson’s ratio, large tensile strain, programmability, and other characteristics [23].

Three-dimensional chiral metamaterials are a class of mechanical metamaterials with special properties, which have received widespread attention in recent years. Overall rotational motion of three-dimensional chiral mechanical metamaterials can be induced through internal deformation rather than external forces, and their global rotational effect can be optimized by adjusting their structural parameters [1]. For example, the twisting behavior of three-dimensional chiral mechanical metamaterials can be adjusted by changing the geometric shape of the helix, which can achieve precise control of the helix structure’s parameters to regulate light transmission [8]. The combination of chiral metamaterials and plasmonic nanostructures can significantly enhance the optical chiral signal of chiral molecules [9]. In addition, as an auxiliary structure, by adjusting the design parameters of chiral metamaterials, negative three-dimensional Poisson’s ratio characteristics can be stably achieved over a large strain range [18].

Coupled compression–torsion effects are attractive characteristics of three-dimensional chiral mechanical metamaterials, and the unique deformation mechanism behind them has attracted widespread attention from researchers. Tobias et al. [11] designed a kind of three-dimensional compression–torsion metamaterial with a cubic unit cell composed of a tetrachiral lattice on each surface. The metamaterial twisted more than 2 degrees per unit axial strain. Zheng et al. [24] proposed a novel three-dimensional tension-torsion metamaterial by connecting adjacent chiral honeycomb layers and inclined rods. This metamaterial exhibited significant changes in torsion angle at a tensile strain of 1% and maintained a high torsion angle even when the number of cells was large. Li et al. [25] proposed a novel compression–torsion three-dimensional metamaterial design method based on the shear–compression coupling effect of two-dimensional materials; they found that infinite torsion angle per axial strain could be achieved through elaborate design, providing new ideas for designing three-dimensional structures with multifunctional applications. Zhong et al. [26] also proposed a novel three-dimensional compression–torsion mechanical metamaterial by converting axial strain into torsional strain based on the inclined rod spiral mechanism. Compared with previously reported three-dimensional compression–torsion metamaterials, this material exhibited superior compression–torsion performance. The factors affecting the compression–torsion characteristics were also discussed in detail, providing guidance for designing three-dimensional metamaterials with excellent compression–torsion characteristics. Kumar et al. [27] investigated the fabrication of a Cu/SiC surface composite via friction stir processing (FSP) for heat sink applications. By embedding SiC particles into a copper matrix using FSP—a solid-state technique—uniform particle distribution was achieved in the composite without defects like porosity or clustering. The material exhibited a 57% increase in hardness and a 13% improvement in tensile strength compared to pure copper, although ductility decreased due to grain refinement and reinforcement effects. The enhanced wear resistance was attributed to SiC’s hardness and homogeneous dispersion. The results highlight FSP’s potential for producing high-performance thermal materials with balanced mechanical and tribological properties. Fang X et al. [28] presented a chiral mechanical metamaterial based on twist buckling, which significantly enhanced elastic energy storage density by coupling axial compression with torsional deformation through freely rotatable metacell structures. Compared to conventional non-chiral designs, this material achieved 5–10 -higher buckling strength and 2–160× greater energy density as well as supported recoverable strains up to 20% while maintaining high stiffness. The key mechanism was in storing additional energy via torsional deformation without substantially increasing local stress concentrations. Experimental validation using 3D-printed prototypes confirmed its superior performance, demonstrating potential applications in lightweight energy storage, impact-resistant structures, and low-frequency vibration isolation.

However, it should be noted that due to the complexity of geometric models and physical mechanisms, research on three-dimensional chiral mechanical metamaterials is still limited. Most studies have analyzed their quasi-static mechanical behavior and deformation process, and research on their dynamic characteristics deserves more attention. The coupled effects of compression–torsion under dynamic loading conditions are still unclear. In addition, the close relationship between the coupled compression–torsion effect and the size effect is significantly affected by the array number of unit cells. Therefore, investigating the dynamic influence mechanism of the size effect on compression–torsion deformation is also a very interesting scientific problem.

In this paper, the quasi-static and dynamic compression mechanical behavior of three-dimensional chiral mechanical metamaterials is investigated. The effects of geometric parameters and size on the coupled compression–torsion effect (i.e., twist angle per axial strain and effective modulus) are studied. The rest of this paper is organized as follows: Section 2 describes the structural and computational model, and the simulation method is validated in Section 3. The quasi-static compression behavior and dynamic compression behavior are discussed in Section 4 and Section 5, respectively. Finally, the key conclusions are summarized in Section 6.

## 2. Materials and Methods

**Figure 1:**
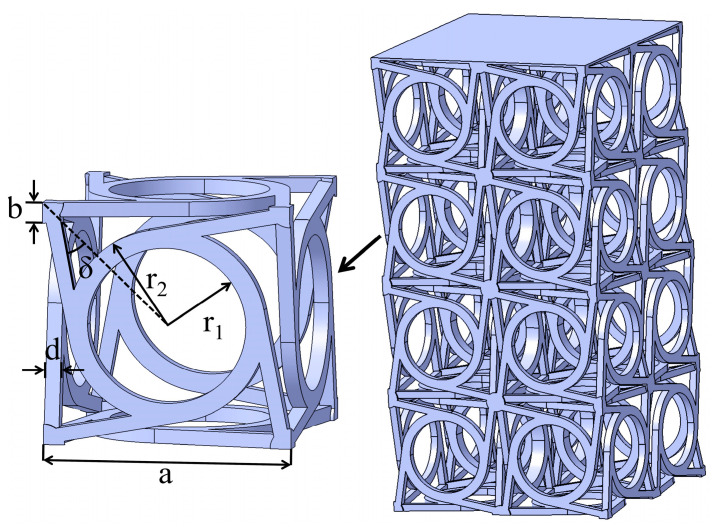
The unit cell as well as structural pattern of three-dimensional chiral mechanical metamaterials. The corresponding geometric parameters, including the unit cell size a, the angle δ, the inner radius r_1_, the outer radius r_2_, the corner width b, and the ligament width d, are all marked.

In Figure 1, the unit cell configuration as well as structural pattern of the three-dimensional chiral mechanical metamaterials are illustrated. This paper is based on the linear elasticity eigenstructure model ($\sigma_{ij}=C_{ijkl}\varepsilon_{kl}$) and explicit kinetic equations ($\rho {\ddot{u}}_{i}=\nabla \cdot \sigma_{ij}+f_{i}$) that model the dynamic compression process. The unit cell configuration and equations were proposed in [11], where the target structure was generated by arraying the corresponding unit cell in three orthogonal directions. Geometric parameters, including the unit cell size a, the angle δ, the inner radius r_1_, the outer radius r_2_, the corner width b, and the ligament width d, are also all indicated in the figure. It should be noted that a=LN, where *N* is the number of arrays.

To simulate its dynamic compression mechanical behavior and gain insight into the effects of size, commercial finite element software ABAQUS 2020/Explicit (Dassault Systèmes, France) was utilized. The linear elastic–plastic mechanical behavior of the constituent material was assumed [11], which was used to simplify the calculation, and the impact of simplification was ignored particularly for the dynamic behavior, with a density of 1.15 g/cm^3^, Young’s modulus of 2.6 GPa, Poisson’s ratio of 0.4, and yield stress of 69 MPa. To ensure the degrees of freedom in rotational deformation, the number of arrays in the Z direction was set as 2N. The bottom side of the specimen was fixed on a rigid substrate, and the top side, made of the same constituent material, was connected to a square plate with a thickness of 10 µm. General contact with a penalty friction coefficient of 0.3 was applied between the target structure and the rigid plates. The structural pattern was meshed using modified quadratic tetrahedral elements (C3D10M) with an element size of 0.015 mm, and the reliability of the calculated results was ensured through mesh convergence analysis. The boundary between quasi-static and dynamic compression was delineated by the strain rate, which ranged from 10^−5^ to 10 s^−1^ for quasi-static compression to 10–10^3^ s^−1^ for low-speed dynamic compression. This paper focuses on the study of three-dimensional chiral mechanical metamaterials under quasi-static and low-speed dynamic compression. In order to simulate the mechanical behavior of three-dimensional chiral metamaterials under static and dynamic compressive loading, the quasi-static mechanical behavior simulation loading method was used to control the displacement loading method, and the dynamic mechanical behavior simulation loading method was used to control the velocity loading method. The calculation time of the simulation was 0.01 s, and the dynamic loading rate was equal to the total strain/time period. The strain rate for quasi-static compression was 10^−1^ s^−1^. Depending on the amount of deformation, the speed of dynamic loading was 15 mm/s, 30 mm/s, 45 mm/s, 60 mm/s, or 75 mm/s.

## 3. Simulation Method Validation

**Figure 2:**
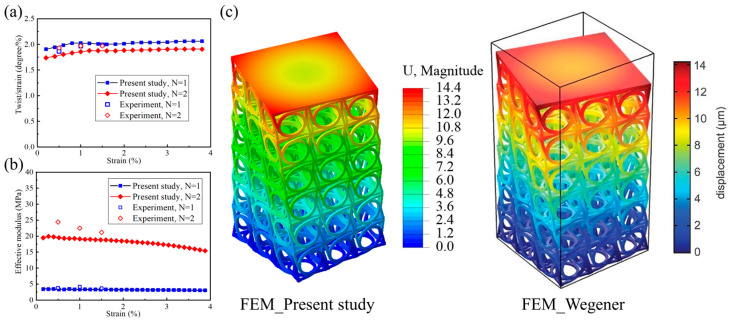
Comparison of (**a**) twist angle per axial strain and (**b**) effective compression modulus versus applied axial strain between the present study and the experiment in [11]. (**c**) Comparison between the displacement vector field obtained in [11] and that obtained in this study.

To validate the accuracy of the computational model, the numerical results were compared with the experimental data and the displacement vector field from [11]. As shown in Figure 2, the twist angle per axial strain and effective compression modulus versus the applied axial strain of the target structure with N = 1 and N = 2 were both analyzed. It can be observed that the variations in the simulated curves obtained in this article were consistent with the experimental data points. Additionally, the simulated displacement vector field also matched well with the calculation results from the chiral micropolar continuum mechanics [11]. The comparisons of the data points and distribution of the displacement field demonstrated the effectiveness of the calculation results in this study. It should be noted in particular that this study ignored the nonlinear behavior of the material (e.g., plasticity and viscoelasticity), a simplification that reduced computational complexity but could have led to biased predictions of local stress concentrations or energy dissipation mechanisms under dynamic loading. Although actual materials may yield at larger strains, the results in this paper are only applicable to mechanical behavior in the elastic range. Future work is needed to verify the critical conditions at the elastic–plastic boundary in conjunction with experiments (Figure 2).

## 4. Discussion

### 4.1. Investigation of Geometric Parameters

The unit cell is an important constituent element of structural materials, and its mechanical properties can be affected by geometric dimensions. Thus, exploring the effects of key geometric parameters on the mechanical behavior of the unit cell is of great significance. Compared to Zhong et al., our study showcases the effect of geometric parameters including the outer radius and corner width on the coupled compression–torsion effects on the three-dimensional chiral mechanical metamaterial in [11]. This section mainly focuses on the effects of the outer radius $r_{2}$ and corner width b on the twist angle per axial strain and effective modulus. The effects of the other parameters are not critical, and thus they are not considered in this article.

#### 4.1.1. The Effect of the Ratio of the Outer Radius on the Unit Cell Size

**Figure 3:**
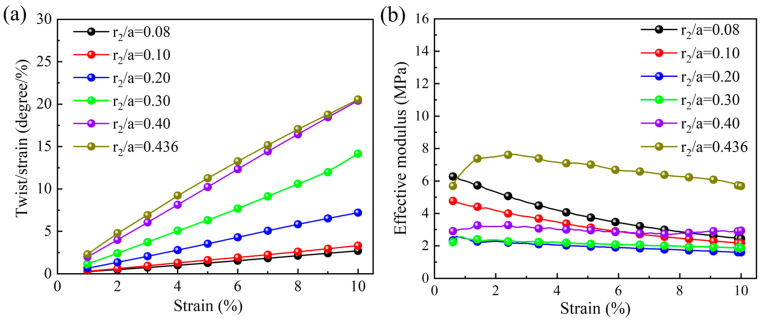
Effect of $r_{2}/a$ under quasi-static compression loading: (**a**) relationship between twist angle per axial strain and applied axial strain; (**b**) relationship between effective modulus and applied axial strain.

In Figure 3a, the relationship between the quasi-static compression twist angle per axial strain and the applied axial strain under different $r_{2}/a$ values is plotted. Herein, $r_{2}/a=0.436$ corresponds to a limit state of the three-dimensional chiral mechanical metamaterials, where the central ring and the ligament are in contact and tangent with each other. It can be observed that the twist angle per axial strain increases with the increase in the applied axial strain, almost in a linear growth trend. Additionally, when the specific axial compression strain is considered, the twist angle per axial strain also increases with the increase in the ratio of the outer radius to the unit cell size. Particularly, when the axial compression strain is 0.1, the twist angle per axial strain is 20.55 degrees per unit strain for the $r_{2}/a=0.436$ pattern and is 2.69 degrees per unit strain for $r_{2}/a=0.08$. The corresponding relationship between the effective modulus and the applied axial strain is given in Figure 3b. As the applied axial strain increases, the effective modulus shows a slow decreasing trend. However, different from the variation in the twist angle, the effective modulus shows a trend of first decreasing and then increasing with the increase in $r_{2}/a$. The maximum value of the effective modulus of 7.61 MPa can be attained when $r_{2}/a=0.436$. The possible reason why the effective modulus increases at higher $r_{2}/a$ is that the central ring and the ligament are in contact and tangent with each other.

#### 4.1.2. The Effect of the Ratio of Corner Width to the Unit Cell Size

**Figure 4:**
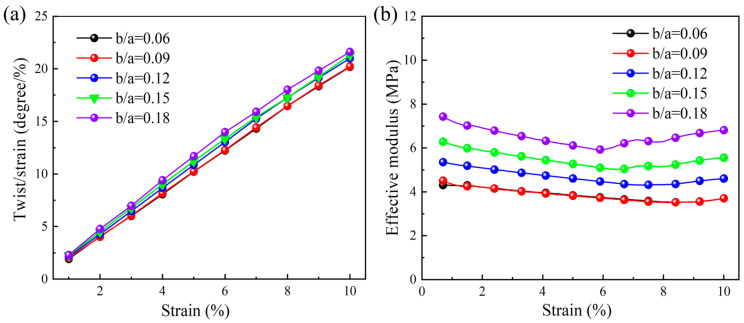
Effect of b/a under quasi-static compression loading: (**a**) relationship between twist angle per axial strain and applied axial strain; (**b**) relationship between effective modulus and applied axial strain.

Figure 4a shows the quasi-static compression relationship between the twist angle per axial strain and the applied axial strain. It can be seen that the quasi-static compression twist angle per axial strain increases with the applied axial strain for all b/a values. When b/a ranges from 0.06 to 0.18, the twist angle per axial strain also shows a gradually increasing trend, but the growth rate is very small. When the quasi-static compression strain is 0.1, the maximum twist angle per axial strain is 21.61 degrees per unit strain, and the minimum value is 20.19 degrees per unit strain. The quasi-static relationship between the effective modulus and the applied axial strain is indicated in Figure 4b. Overall, the effective modulus decreases slightly with the increase in the axial strain. The maximum effective modulus of 7.43 MPa is achieved when b/a=0.18, and the minimum effective modulus of 3.69 MPa is obtained when b/a=0.06.

### 4.2. Investigation of Size Effect

Size has a significant effect on the mechanical properties and deformation behavior of cellular materials, and exploring the underlying rules is an interesting topic. Different from Zhong et al. and Frenzel et al., our research expanded the stacking method of unit cells and analyzed the influence of size on the coupled compression–torsion effect of three-dimensional chiral metamaterials in more detail.

Herein, the effects of the cell stacking method and size on the quasi-static coupled compression–torsion characteristics and effective elastic modulus were investigated. A basic structure with characteristic dimensions of L=500 μm, unit cell size a=LN, ligament width d=0.06a, corner width $b=\sqrt{2}d$, inner radius $r_{1}=0.32a$, and outer radius $r_{2}=0.4a$, was selected and considered.

#### 4.2.1. The Effect of the Number of Arrays in the X and Y Directions

**Figure 5:**
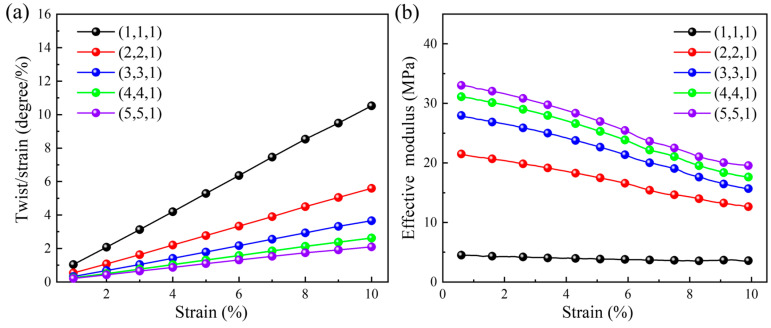
The effect of the number of arrays in the X and Y directions under quasi-static compression loading: (**a**) the relationship between the twist angle per axial strain and the applied axial strain; (**b**) the relationship between the effective modulus and the applied axial strain.

Figure 5a illustrates the variation between the quasi-static compression twist angle per axial strain and the applied axial strain under different number of arrays in the X and Y directions. It should be noted that, when exploring the effects of the number of arrays in the X and Y directions, the number of arrays in the Z direction remains unchanged (i.e., kept as 1) and is annotated in the legend. The quasi-static compression twist angle per axial strain increases with the increase in the applied axial strain. However, a smaller twist angle per axial strain is obtained for a higher array number in the X and Y directions. The reason for this phenomenon is the size effect. The larger the array number in the X and Y directions, the stronger the ability of three-dimensional chiral mechanical metamaterials to resist torsion, and the corresponding twist angle per axial strain is smaller. When the axial compression strain is 0.1, the maximum twist angle per axial strain is 10.52 degrees per unit strain for the structural pattern with one horizontal array, and the minimum twist angle per axial strain is 2.09 degrees per unit strain for five horizontal arrays. Correspondingly, the relationship between the quasi-static compression effective modulus and the applied axial strain under different number of arrays in the X and Y directions is given in Figure 5b. As the compression progresses, effective modulus exhibits a gradual downward trend. A higher effective modulus, i.e., higher stiffness or a stronger ability to resist deformation, is obtained with larger array numbers in the X and Y directions. Thus, the twist angle per axial strain exhibits a downward trend, which is consistent with those shown in Figure 5a. When the compression strain is 0.1, the maximum and minimum moduli are 19.52 MPa and 3.59 MPa, respectively.

#### 4.2.2. The Effect of the Number of Arrays in the Z Direction

**Figure 6:**
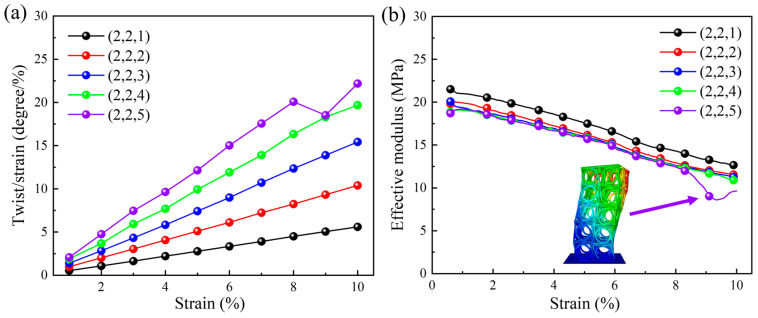
The effect of the number of arrays in the Z direction under quasi-static compression loading: (**a**) the relationship between the twist angle per axial strain and the applied axial strain; (**b**) the relationship between the effective modulus and the applied axial strain.

The relationship between the quasi-static compression twist angle per axial strain and the applied axial strain for different numbers of arrays in the Z direction is plotted in Figure 6a. The number of unit cells in the X and Y directions remains constant (i.e., kept as two), while the number of unit cells in the Z direction varies between one and five. When the applied axial strain increases, the twist angle per axial strain also shows an increasing trend. The quasi-static compression twist angle per axial strain increases with the increase in the number of arrays in the Z direction, owing to the decrease in the ability to the resist torsion caused by the size effect. When the compression strain is 0.1, the maximum twist angle per axial strain is 22.18 degrees per unit strain, and the minimum value is 5.59 degrees per unit strain. On the contrary, when the number of arrays in the Z direction is five, a sudden drop emerges under large compressive strain. It results from the instability in the structural materials during compression. The variation in effective modulus is shown in Figure 6b, where a decreasing trend can be observed as the array number in the Z direction increases. Similarly, when the array number in the Z direction is five, a sudden drop is also observed in the effective modulus, which is consistent with the phenomenon shown in Figure 6a. In this case, effective modulus is only 8.29 MPa, which is caused by the instability of the three-dimensional chiral mechanical metamaterials due to the shift in the center of gravity (see the instability deformation diagram in Figure 6b).

## 5. Dynamic Compression Behavior

The above sections discussed the effects of the geometric parameters and size on the quasi-static compression behavior of three-dimensional chiral mechanical metamaterials. The insight into the effects of size on the dynamic mechanical behavior is more complex and interesting. Thus, in the following section, the effects of size (i.e., the array number in the horizontal and vertical directions) on the dynamic coupled compression–torsion characteristics and effective elastic modulus are investigated.

### 5.1. Size Effects in the X and Y Directions

**Figure 7:**
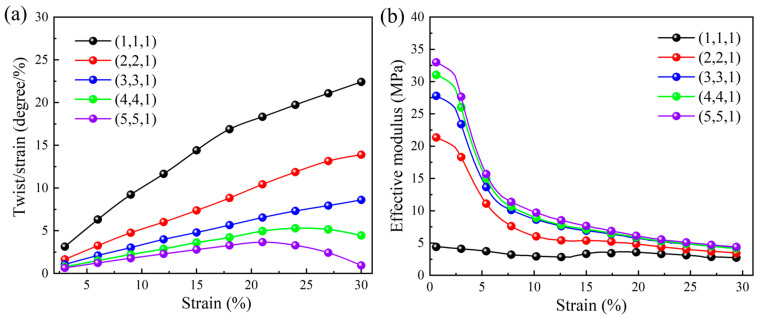
The effect of the number of arrays in the X and Y directions under dynamic compression loading: (**a**) the relationship between the twist angle per axial strain and the applied axial strain; (**b**) the relationship between the effective modulus and the applied axial strain.

In Figure 7a, the relationship between the dynamic compression twist angle per axial strain and the applied axial strain under different number of arrays in the X and Y directions is illustrated. When the number of arrays in the X and Y directions increases, the ability of three-dimensional chiral mechanical metamaterials to resist torsion also increases, and the corresponding twist angle per axial strain shows a decreasing trend. When the array number in the X and Y directions is four or five, the dynamic compression twist angle per axial strain increases first and then decreases. This is due to the mutual strain between ligaments during the twist of the unit cells, which hinders the freedom of rotation. When the dynamic compression strain is 0.3, the maximum twist angle per axial strain is 22.41 degrees per unit strain, and the minimum value is 0.93 degrees per unit strain. Correspondingly, the dynamic compression effective modulus exhibits an increasing trend as the array number in the X and Y directions increases (see Figure 7b), indicating an increase in the structural stiffness, which is also consistent with the results shown in Figure 7a. When the compression strain is 0.3, the maximum and minimum dynamic compression effective moduli are 4.30 MPa and 2.70 MPa, respectively.

Furthermore, we compare the dynamic compression results with those obtained under quasi-static loading conditions for different numbers of arrays in the X and Y directions. Under specific axial compression strain, the values of the twist angle per axial strain under static and dynamic compression are almost the same, but the effective modulus decreases more sharply under the dynamic loading condition. From another perspective, it can be seen that under the dynamic loading condition, even if the effective modulus decreases more, the twist angle per axial strain under the same compressive strain remains almost unchanged, which may be due to inertia.

### 5.2. Size Effects in the Z Direction

**Figure 8:**
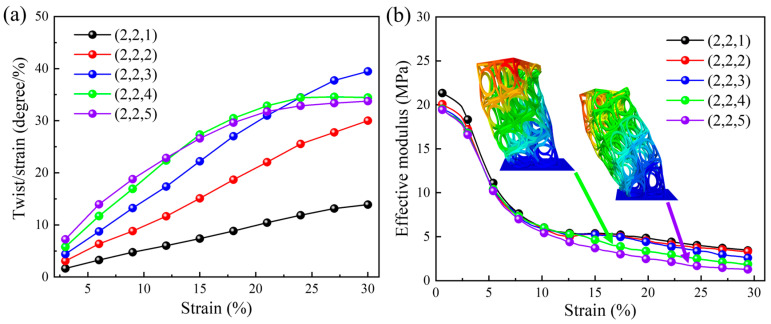
The effect of the number of arrays in the Z direction under dynamic compression loading: (**a**) the relationship between the twist angle per axial strain and the applied axial strain; (**b**) the relationship between the effective modulus and the applied axial strain.

Figure 8a illustrates the relationship between the dynamic compression twist angle per axial strain and the applied axial strain for different numbers of arrays in the Z direction. As the applied dynamic compression axial strain increases, the overall twist angle per axial strain shows an increasing trend. Additionally, a larger twist angle per axial strain can be obtained with a larger array number in the Z direction. The ability of structural materials to resist torsion decreases as the array number in the Z direction increases. However, when the number of arrays is four or five, buckling instability occurs during the dynamic compression process, resulting in a slower increase in the twist angle per axial strain. When the dynamic compression strain is 0.3, the twist angle per axial strain is 33.76 degrees per unit strain for the structural pattern with five vertical arrays and is 13.88 degrees per unit strain for the structural pattern with one vertical array. Figure 8b shows the relationship between the dynamic compression effective modulus and the applied axial strain. When the array number in the Z direction increases, the stiffness, i.e., the effective elastic modulus, of the structural material decreases. Thus, structures with fewer arrays in the vertical direction have a higher effective modulus, and the corresponding twist angle per axial strain is smaller. When the dynamic compressive strain is 0.3, the maximum and minimum effective moduli are 3.4 MPa and 1.26 MPa, respectively. The effective modulus of structures with four or five arrays in the Z direction is significantly lower than that of structures with other numbers of arrays. This results from the buckling instability of the struts (see the instability deformation diagram in Figure 8b).

The static and dynamic results under different numbers of arrays in the Z direction are analyzed and compared. Similar patterns are shown in Section 5.1. The values of the twist angle per axial strain under static and dynamic compression are almost the same, but the effective modulus decreases more sharply under the dynamic loading condition. The reason for this phenomenon may be inertia.

## 6. Conclusions

In this paper, the quasi-static and dynamic compression mechanical behavior of three-dimensional chiral mechanical metamaterials was investigated. The effects of geometric parameters and size on the twist angle per axial strain and effective modulus were discussed. The key conclusions gained from this study were as follows:

(1) Under the quasi-static compression loading condition, the twist angle per axial strain increases with the increase in the applied axial strain, while the effective modulus shows a decreasing trend. The twist angle per axial strain increases with the increase in the ratio of the outer radius to the unit cell size or the ratio of the corner width to the unit cell size.

(2) Under the quasi-static compression loading condition, a smaller twist angle per axial strain and a higher effective modulus can be obtained with larger array number in the X and Y directions. When the array number in the Z direction increases, the twist angle per axial strain exhibits an increasing trend, while a decreasing trend is observed in the effective modulus.

(3) Under the dynamic compression loading condition, when the number of arrays in the X and Y directions increases, the twist angle per axial strain shows a decreasing trend, while the effective modulus under dynamic compression exhibits an increasing trend. A larger twist angle per axial strain and a lower effective elastic modulus can be obtained with larger array numbers in the Z direction.

(4) Comparing the quasi-static and dynamic compression results shows that the effective modulus and twist angle per axial strain are not linearly related to the strain, and the effective modulus decreases more drastically under the dynamic loading condition, which may be due to inertia.

This study systematically revealed the mechanical behavior of 3D chiral mechanical metamaterials under quasi-static and dynamic compressive loading and its key regulatory mechanisms. The significant influences of the geometrical parameters and size on the properties of compression–torsion coupling were clarified through numerical simulations, and the dominant role of inertia on the effective modulus under dynamic loading was elucidated. It was found that the effective modulus decreases drastically under dynamic compression due to the effects of inertia, while the torsion angle is more consistent with the quasi-static results, which extends the traditional quasi-static theory and provides new insights for structural design under dynamic loading. In addition, an increase in the number of arrays in the X/Y directions suppresses torsional deformation and enhances stiffness, while the opposite occurs in the Z direction, revealing the anisotropic characteristics of the size effect. The results fill the knowledge gap regarding the dynamic compression–torsion coupling mechanism and provide theoretical support for engineering applications such as energy absorption and flexible sensing through providing a parametric design framework. Future research can focus on the following directions: (1) analyzing high-strain-rate experiments and microscopic inertia mechanisms in depth; (2) optimizing the performance of multi-material systems and additive manufacturing processes; (3) cross-scale modeling of coupled thermal–force–electrical multi-field behaviors; and (4) developing prediction methods for dynamic failure thresholds and non-equilibrium state responses.

## Data Availability

Data will be made available upon request.
